# Association of altered fibrinogen indexes levels as a potential biomarker in determining the possible onset of diabetic retinopathy

**DOI:** 10.1038/s41598-023-50738-5

**Published:** 2023-12-27

**Authors:** Yong Zhuang, Qingyan Cai, Xin Hu, Huibin Huang

**Affiliations:** Department of Endocrinology, The Second Affiliated Hospital of Fujian Medical University, No.950 Donghai Street, Fengze District, Quanzhou City, 362000 Fujian Province China

**Keywords:** Biomarkers, Diseases, Endocrinology, Medical research

## Abstract

Research suggests that fibrinogen was related to diabetic retinopathy (DR). Then, the relationship between functional indices of fibrinogen and detailed staging of DR has not been explored. Type 2 diabetic and healthy control subjects (n = 960) were recruited in a cross-sectional study. Participants with type 2 diabetes mellitus were categorized into five stages according to their fundus lesions, and fibrinogen (Fib) and its functional indices (angle α and k value) were measured. The angle α levels increased in diabetic subjects with retinopathy compared with those without, and it was significantly elevated early in retinopathy. In contrast, the k value levels slightly decreased. Despite observing an increase in angle α levels and a decrease in k value levels during the later stages of retinopathy compared to the earlier stages, there was no statistically significant difference in the later stages. The association of the angle α and k value with DR was independent of the hyperglycaemic state and other potential confounders (OR = 1.672, 95% CI 1.489–1.876, *P* < 0.01; OR = 0.013, 95% CI 0.004–0.041, *P* < 0.01). The angle α levels and k value levels were closely correlated with retinopathy (*r* = 0.593, *P* < 0.00; *r* = − 0.646, *P* < 0.01). The ROC curve indicated that the diagnostic value of angle α and k value were (AUC = 0.897, *P* < 0.001; AUC = 0.859, *P* < 0.001). Fibrinogen function indexes, such as angle α and k value, may be valuable for the early diagnosis of DR but do not directly assess the severity of DR.

## Introduction

It has been acknowledged that the global prevalence of diabetes mellitus is increasing yearly, with the number of adults with diabetes reaching 537 million cases worldwide in 2021^1,2^. Diabetic retinopathy (DR) is a common chronic complication of diabetes mellitus, with a prevalence of 35.4%^3^, and about 8% of patients become blind, which seriously affects the quality of life. Early typical symptoms of DR are not significant and easily overlooked, and the progression of the disease can lead to adverse outcomes such as blindness. The current clinical methods for diagnosing DR are optical coherence tomography (OCT), fundus examination, fundus angiography, wide-angle imaging systems, and even artificial intelligence^4^. Still, the examinations are time-consuming, labor-intensive, and have limitations due to the high equipment cost. There are few simple and feasible methods for diagnosing DR, with limited use of serum markers and even fewer studies dedicated to early-stage DR. Finding a simple and easy-to-use detection method is, therefore, significant.

The pathogenesis of DR remains uncertain, but it is believed to involve the functional impairment of retinal microvascular smooth muscle cells and endothelial cells, abnormal fibrinolytic function, and inflammation^5,6^. Fibrinogen (Fib) levels increase in the presence of endothelial dysfunction, hypercoagulability, or inflammation. Studies^7^ have established a significant association between Fib, an indicator of coagulation function, and DR. Fib is a glycoprotein produced and released by hepatocytes. It acts as a substrate for thrombin and plays a role in coagulation. Moreover, it can serve as an indicator of the body’s inflammatory status^8^. The conventional Fib assay only measures the quantity of Fib in the blood, providing no assessment of its functional capacity. Angle α and k value primarily indicate Fib function. In our previous study, we discovered a significant correlation between angle α, k value, and the presence of DR.

In this study, building upon our previous research, we increased the sample size. We conducted a detailed categorization of participants with diabetic retinopathy (DR) to explore the correlation between angle α, k value, and different stages of DR.

## Methods

### Participants information

960 participants were enrolled in this study at the Second Affiliated Hospital of Fujian Medical University from 2019 to 2022. Among them, 803 individuals met the 1999 World Health Organization (WHO) type 2 diabetes diagnostic criteria. Additionally, 157 subjects from the hospital's health examination population were selected as healthy controls during the same period. The exclusion criteria were diabetic ketosis, age < 25 years or > 75 years, glaucoma, ocular trauma, trauma surgery, pregnancy, kidney or liver insufficiency, perioperative, severe infection, neoplasm, and blood disease. Participants continued to use their previous hypoglycemic medications or insulin regimens; additionally, they continued to use antihypertensive drugs and lipid-lowering agents if necessary. All participants were informed and signed consent forms. The Ethics Committee of the Second Affiliated Hospital of Fujian Medical University has approved the study.

### Indicator measurement

Each participant was tested by the same physician with the same experience following standard procedures. All inspections and tests were carried out in a comfortable and quiet laboratory. All patients underwent electromyography (EMG) using the Keypoint 9033A07 instrument from Denmark. On the same one-piece scale, standing height and weight were measured under fasting without shoes. The BMI value was obtained by calculating the participant’s weight (kg) divided by the square of height (m)^2^. The blood pressure of the participant's right arm was measured with a mercury sphygmomanometer after 30 min of sitting and resting. Venous blood was collected from each participant in the morning after a 10–12 h fast. The angle α and k value were determined using a thromboelastography analyzer (CFMS LBPU-8800; Lepu, Beijing). Fib levels were measured using a blood coagulation meter (FAC21A-UW; Ltd, Taiwan). We used high-performance liquid chromatography to detect HbA1c (D10; Bio–Rad, Berkeley, CA). We used automatic biochemical analyzers (Cobas 8000; Roche, Germany) uniformly to measure fasting plasma glucose, liver function [aspartate transaminase (AST), alanine transaminase (ALT)], kidney function (serum creatinine), and blood lipids [low-density lipoprotein (LDL) cholesterol, high-density lipoprotein (HDL) cholesterol and total cholesterol (TC)]. Urinary albumin concentration and urinary creatinine were evaluated using immunohypertensive assays. This study was calculated from the ratio of urinary albumin (mg) to urinary creatinine (g) urinary albumin-creatinine ratio (UACR). All glomerular filtration rates were obtained by the formula “Ccr = {[140-age (years) × weight (kg)]/[0.818 × serum creatinine (Scr, µmol/L)]} for men and Ccr × 0.85 for women”.

### Diagnosis and stages of retinopathy

An ophthalmic technician examined all subjects for visual acuity and fundus examination (fundus imaging was performed when fundus examination was not definitive). The diagnosis was made by senior ophthalmologists and staged according to the Diabetic Retinopathy Disease Severity Scale^9^. Stage 1 was defined as No apparent retinopathy. Stage 2 was defined as Mild nonproliferative diabetic retinopathy. Stage 3 was defined as Moderate nonproliferative diabetic retinopathy. Stage 4 was defined as Severe nonproliferative diabetic retinopathy. Stage 5 was defined as Proliferative diabetic retinopathy (Table 1).

**Table 1:** Diabetic retinopathy disease severity scale.

Stage	Proposed disease severity level
1	No apparent retinopathy
2	Mild nonproliferative diabetic retinopathy
3	Moderate nonproliferative diabetic retinopathy
4	Severe nonproliferative diabetic retinopathy
5	Proliferative diabetic retinopathy

### Statistical analysis

We used SPSS version 19.0 for Windows (SPSS Inc., Chicago, IL) software for statistical analysis. The data were expressed as the mean (standard deviation, SD) for normally distributed data. Non-normally distributed variables were tested with nonparametric tests. The count data were compared using the chi-square test. The multiple comparisons among groups were assessed using ANOVA for variables. Angle α and k value were added to the logistic regression model to control for possible confounders. The relation of the angle α and k value levels to the stages of retinopathy was calculated using Spearman’s correlation analysis. An ROC curve was conducted with MedCalc Software version 15.2 to assess the accuracy of serum angle α levels and k value levels in distinguishing between patients with and without diabetic retinopathy. The optimal cut-off point was identified by calculating the area under the curve (AUC). *P* < 0.05 was considered statistical significance.

### Ethics approval and consent to participate

This study was performed in line with the principles of the Declaration of Helsinki. Approval was granted by the Ethics Committee of the Second Afliated Hospital of Fujian Medical University (Date 2020/No302). Informed consent was obtained from all individual participants included in the study. All volunteers agreed and signed informed consent.

## Results

**Figure 1:**
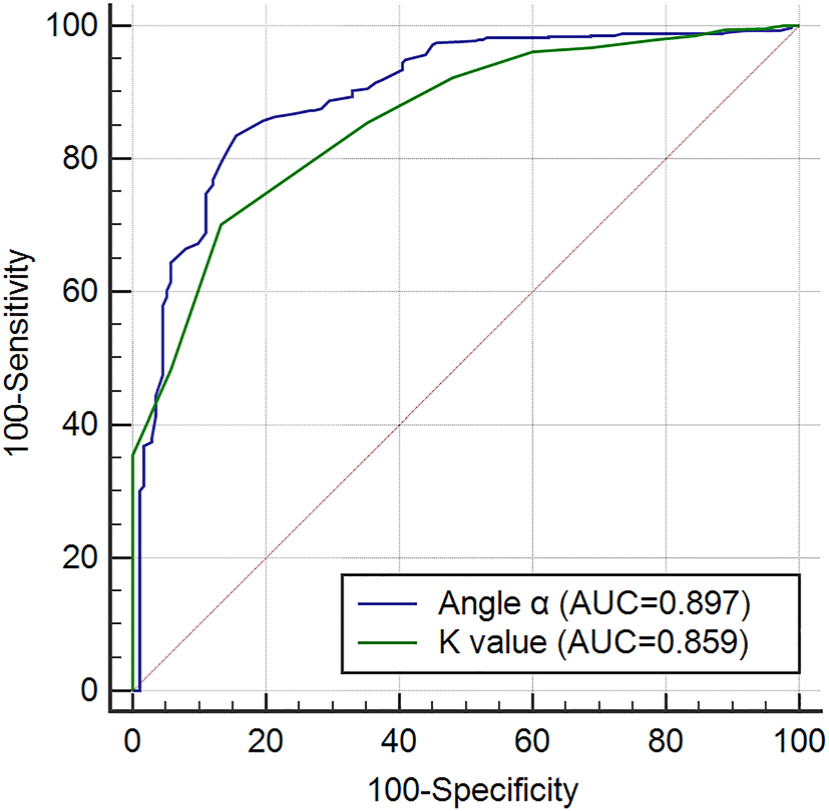
ROC curve of angle α and k value.

The study involved 960 participants aged between 25 and 75 years. Among them were 157 healthy control subjects and 803 individuals with diabetes, categorized into different stages: 173 in stage 1, 161 in stage 2, 158 in stage 3, 160 in stage 4, and 151 in stage 5 (Table 2). Among the six groups of subjects, there were no differences regarding sex ratio, age, smoking history, BMI, blood pressure (DBP and SBP), FPG, LDL, HDL, TC, AST, ALT, and rate of diabetic neuropathy. No significant differences were observed in the use of antidiabetic medications, antihypertensive medications, or lipid-modulating medications among participants with diabetes. Subjects with retinopathy had a longer duration of diabetes than those without retinopathy. There was an increase in Fib levels, UACR, and glycated hemoglobin and a decrease in eGFR in the stage 3, 4, and 5 groups. The angle α levels increased significantly at stage 2 and further at stage 3, but there were no significant differences in the comparison between the three groups at stages 3, 4, and 5. The k value levels declined significantly at stage 2, declined further at stage 3, and were more pronounced at stage 4, but there was no significant difference in the comparison between the two groups at stages 4 and 5 (Table 3). The angle α and k value levels in relation to retinopathy was further assessed in a multivariate model. After adjustment, the angle α and k value were still independently associated with DR (odds ratio 1.672 [1.489–1.876], *P* < 0.001; odds ratio 0.013 [0.004–0.041], *P* < 0.001; respectively) (Table 4). Correspondingly, angle α levels were positively correlated with DR (*r* = 0.593, *P* < 0.001), and k value levels were negatively correlated with DR (*r* =  − 0.646, *P* < 0.001) (Table 5). ROC curve analysis revealed that the optimal threshold values for angle α and k value, which differentiate the presence and absence of DR, were determined to be 60.0 degrees (with a sensitivity of 83.5%, specificity of 84.4%, AUC = 0.897, *P* < 0.001) and 1.8 min (with a sensitivity of 70.2%, specificity of 86.7%, AUC = 0.859, *P* < 0.001); respectively (Fig. 1).

**Table 2:** Comparison of clinical features between different groups.

Group	Healthy volunteer	Stage 1	Stage 2	Stage 3	Stage 4	Stage 5	*P* value
Case (male/female)	88/69	92/81	90/71	85/73	88/72	81/70	0.992
Age (years)	51.1 ± 8.2	50.7 ± 9.0	51.8 ± 7.9	51.9 ± 7.1	50.7 ± 7.6	50.6 ± 8.6	0.527
DM duration (years)	–	6.6 ± 3.7	7.4 ± 3.1	7.8 ± 2.8	8.6 ± 3.6	8.6 ± 3.6	< 0.01
Smoking (N/Y)	116/41	133/40	111/50	109/49	110/50	113/38	0.385
SBP (mmHg)	122 ± 5	125 ± 7	124 ± 8	125 ± 8	126 ± 9	125 ± 9	0.709
DBP (mmHg)	77 ± 6	76 ± 8	77 ± 7	77 ± 8	76 ± 9	76 ± 9	0.554
BMI (kg/m ^2^)	24.3 ± 2.5	23.9 ± 2.1	24.5 ± 1.9	23.8 ± 2.2	23.7 ± 1.8	24.6 ± 52	0.192
FPG (mmol/L)	4.7 ± 0.5	8.4 ± 1.3	8.2 ± 1.6	8.5 ± 1.8	8.3 ± 1.7	8.2 ± 1.7	< 0.01
HbA1c (%)	4.8 ± 0.5	7.9 ± 1.3	8.5 ± 1.5	8.8 ± 1.9	8.6 ± 1.4	8.6 ± 1.5	< 0.01
TC (mmol/L)	4.8 ± 0.9	4.9 ± 0.9	4.9 ± 0.7	5.0 ± 0.9	4.9 ± 0.8	4.8 ± 1.0	0.221
LDL-C (mmol/L)	2.8 ± 0.8	2.9 ± 1.1	2.8 ± 0.9	3.0 ± 1.1	2.9 ± 0.9	2.8 ± 1.1	0.218
HDL-C (mmol/L)	1.5 ± 0.7	1.4 ± 0.6	1.3 ± 0.5	1.4 ± 0.7	1.3 ± 0.7	1.4 ± 0.5	0.227
ALT (IU/L)	23 ± 3	23 ± 4	24 ± 3	25 ± 4	24 ± 4	23 ± 5	0.581
AST (IU/L)	22 ± 4	22 ± 5	22 ± 4	23 ± 6	22 ± 4	24 ± 6	0.352
Neuropathy (%)	–	9.2	13.7	15.8	15.0	14.6	0.392
UACR (mg/g)	20.5 ± 2.8	21.6 ± 3.0	28.9 ± 10.6	30.9 ± 15.2	25.7 ± 11.6	26.1 ± 10.6	< 0.01
eGFR [ml/(min·1.73m^2^)]	100.8 ± 33.8	104.9 ± 27.7	102.2 ± 26.8	95.3 ± 20.0	96.4 ± 25.3	95.9 ± 24.8	< 0.01
Fib (g/L)	3.6 ± 0.6	3.7 ± 0.6	3.7 ± 0.6	3.9 ± 0.6	4.3 ± 0.5	4.3 ± 0.5	< 0.01
K value (min)	2.1 ± 0.3	2.1 ± 0.3	1.9 ± 0.2	1.8 ± 0.3	1.6 ± 0.2	1.6 ± 0.2	< 0.01
Angle α (deg)	57.8 ± 2.6	57.5 ± 3.0	60.4 ± 2.3	62.0 ± 2.3	62.5 ± 1.6	62.4 ± 1.7	< 0.01

(Stage 1, No apparent retinopathy. Stage 2, Mild nonproliferative diabetic retinopathy. Stage 3, Moderate nonproliferative diabetic retinopathy. Stage 4, Severe nonproliferative diabetic retinopathy. Stage 5, Proliferative diabetic retinopathy).

**Table 3:** Comparison of k value and angle α between groups.

Stage	k value (min)	Angle α (deg)
1	2.1 ± 0.3	57.5 ± 3.0
2	1.9 ± 0.2	60.4 ± 2.3
3	1.8 ± 0.3	62.0 ± 2.3
4	1.6 ± 0.2	62.5 ± 1.6
5	1.6 ± 0.2	62.4 ± 1.7
*P* value^1^	0.033	< 0.01
*P* value^2^	0.014	< 0.01
*P* value^3^	< 0.01	< 0.01
*P* value^4^	< 0.01	< 0.01
*P* value^5^	< 0.01	< 0.01
*P* value^6^	< 0.01	< 0.01
*P* value^7^	< 0.01	< 0.01
*P* value^8^	< 0.01	0.061
*P* value^9^	< 0.01	0.168
*P* value^10^	0.728	0.638

(^1^*P*, Stage 1 vs. Stage 2. ^2^*P,* Stage 1 vs. Stage 3. ^3^*P*, Stage 1 vs. Stage 4. ^4^*P*, Stage 1 vs. Stage 5. ^5^*P*, Stage 2 vs. Stage 3. ^6^*P*, Stage 2 vs. Stage 4. ^7^*P*, Stage 2 vs. Stage 5. ^8^*P*, Stage 3 vs. Stage 4. ^9^*P*, Stage 3 vs. Stage 5. ^10^*P*, Stage 4 vs. Stage 5).

**Table 4:** Multiple regression analysis of the relation of angle α and k value to DR.

Covariables	*OR*	95% CI	*P* value
Disease course	1.049	0.964–1.143	0.269
FPG	1.039	0.887–1.218	0.636
HbA1c	1.391	1.136–1.703	0.001
Neuropathy	0.811	0.328–2.007	0.651
UACR	1.176	1.104–1.252	< 0.01
eGFR	0.994	0.983–1.004	0.243
Fib	1.407	0.905–2.189	0.129
Angle α	1.672	1.489–1.876	< 0.01
K value	0.013	0.004–0.041	< 0.01

**Table 5:** Correlation analysis between DR and k value and angle α (spearman correlation analysis).

Item	*r*	*P* value
DR		
k value	− 0.646	< 0.01
Angle α	0.593	< 0.01

## Discussion

The results of this study demonstrated that serum angle α and k value are potential biomarkers of DR but do not directly assess the severity of DR. In this study, subjects with DR had higher angle α than those without, and the levels of k value were lower. More importantly, during the early stage of DR (stage 2, mild nonproliferative diabetic retinopathy), changes were observed in both the angle α and the k value. Angle α levels increased and k levels decreased were strongly associated with DR, and this relationship was independent of the covariates. In addition, it is worth noting that the angle α levels increased during stages 3, 4, and 5. However, upon comparing these three periods, no significant difference was observed. Furthermore, the k levels decreased during stages 3, 4, and 5. However, no significant change was observed when comparing stages 4 and 5. It indicated that although angle α and k value levels are altered in the early stages of DR, they cannot be used to assess the severity of DR.

DR is a common microvascular complication of diabetes mellitus and a significant cause of vision loss in the elderly^10^. Due to the lack of knowledge of diabetic patients about diabetic complications, insufficient diabetic health education, and the lack of obvious early symptoms of DR, as well as the low degree of promotion of OCT, fundus angiography, and other detection methods in the clinic, resulting in the majority of diabetic patients failing to carry out regular fundus lesion examination, missing the optimal period of treatment and even blindness. The quest to identify serum markers for early detection of DR is of utmost importance.

The pathogenesis of DR is currently unknown. In the initial stages of diabetic retinopathy, hyperglycemia and altered metabolic pathways lead to oxidative stress and neurodegeneration^11^. Elevated HbA1c levels are significantly associated with the progression of DR^12,13^, consistent with our findings that subjects with DR had significantly elevated HbA1c compared to those without DR. In addition, alterations in proteinuria are closely associated with DR^14^, and our study also found that UACR and eGFR were altered when DR occurred. DR is a retinal microangiopathy that involves alterations in the vessel wall and the rheological properties of the blood. In diabetic patients, due to microvascular endothelial damage, the blood is concentrated in a hypercoagulable state, forming blood clots, and the abnormal coagulation function coupled with other factors such as inflammation is an essential factor in the occurrence of DR^15^. Fib is known to be exacerbated in the presence of a hypercoagulable and inflammatory state within the bloodstream^16,17^. Aris et al.^18^ proposed that reduced fibrinolysis and increased blood viscosity contribute to alterations in hemorheological characteristics in the pathogenesis of DR. Some studies^19^ have shown that diabetic patients with reduced Fib have a concomitant reduction in the incidence of DR. This study also confirmed that subjects with DR had higher Fib levels than individuals without DR. However, this study also found that Fib did not show any changes in the early stages of DR. Fib function indexes, such as the angle α and k value, demonstrate an increase in angle α and a decrease in the k value in a high Fib functional state. In this study, meaningful changes in angle α and k value were observed in the mild nonproliferative DR.

Furthermore, this study determined the optimal cutoff values of angle α and k value levels for distinguishing DR as 60.0 degrees (greater than 62.5 degrees) and 1.8 min (less than or equal to 1.8 min), respectively. Although angle α and k value levels cannot be used to assess the severity of DR, they may provide potential serum biomarkers for early diagnosis of DR. Clinicians can detect DR promptly by monitoring indicators such as angle α, k value, and Fib. When these monitoring indicators show abnormalities, the addition of medications aimed at improving blood viscosity, in addition to diabetes treatment, may play a role in preventing and delaying the occurrence and progression of DR, benefiting patients.

## Conclusions

This study further confirms that coagulation abnormalities are strongly associated with DR. The angle α and k values, as coagulation indicators, may serve as potential biomarkers for the early diagnosis of DR. An altered serum levels of fibrinogen function indexes may indicate that the patient has a possible DR and needs an earlier referral to an ophthalmologist. Still, they cannot be used to assess the severity of DR. However, this study still has some limitations. The study was single-center and mono-ethnic, which may have influenced the results. Further comprehensive studies with larger sample sizes and multiple centers are needed.

## Data Availability

The datasets used or analysed during the current study are available from the corresponding author on reasonable request.
